# Metabolomic Profile at Birth, Bronchiolitis and Recurrent Wheezing: A 3-Year Prospective Study

**DOI:** 10.3390/metabo11120825

**Published:** 2021-11-30

**Authors:** Silvia Carraro, Valentina Agnese Ferraro, Michela Maretti, Giuseppe Giordano, Paola Pirillo, Matteo Stocchero, Stefania Zanconato, Eugenio Baraldi

**Affiliations:** 1 Women’s and Children’s Health Department, University Hospital of Padova, 35128 Padova, Italy; silvia.carraro@unipd.it (S.C.); maretti.mic@gmail.com (M.M.); giuseppe.giordano@unipd.it (G.G.); paola.pirillo@gmail.com (P.P.); matteo.stocchero@unipd.it (M.S.); stefania.zanconato@aopd.veneto.it (S.Z.); eugenio.baraldi@unipd.it (E.B.); 2 Institute of Pediatric Research (IRP), Fondazione Istituto di Ricerca Pediatrica Città della Speranza, 35128 Padova, Italy

**Keywords:** bronchiolitis, recurrent wheezing, metabolomics, urine, neonate

## Abstract

There is growing interest for studying how early-life influences the development of respiratory diseases. Our aim was to apply metabolomic analysis to urine collected at birth, to evaluate whether there is any early metabolic signatures capable to distinguish children who will develop acute bronchiolitis and/or recurrent wheezing. Urine was collected at birth in healthy term newborns. Children were followed up to the age of 3 years and evaluated for the development of acute bronchiolitis and recurrent wheezing (≥3 episodes). Urine were analyzed through a liquid-chromatography mass-spectrometry based untargeted approach. Metabolomic data were investigated applying univariate and multivariate techniques. 205 children were included: 35 had bronchiolitis, 11 of whom had recurrent wheezing. Moreover, 13 children had recurrent wheezing not preceded by bronchiolitis. Multivariate data analysis didn’t lead to reliable classification models capable to distinguish children with and without bronchiolitis or with recurrent wheezing preceded by bronchiolitis neither by PLS for classification (PLS2C) nor by Random Forest (RF). However, a reliable signature was discovered to distinguish children who later develop recurrent wheezing not preceded by bronchiolitis, from those who do not (MCCoob = 0.45 for PLS2C and MCCoob = 0.48 for RF). In this unselected birth cohort, a well-established untargeted metabolomic approach found no biochemical-metabolic dysregulation at birth associated with the subsequent development of acute bronchiolitis or recurrent wheezing post-bronchiolitis, not supporting the hypothesis of an underlying predisposing background. On the other hand, a metabolic signature was discovered that characterizes children who develop wheezing not preceded by bronchiolitis.

## 1. Introduction

Even if the association between acute bronchiolitis and subsequent recurrent wheezing is well known [1,2], the mechanism by which this may occur is poorly understood.

There is an historical debate whether the viral acute infection causes the subsequent recurrent wheezing, by affecting airway structure or by inducing a long-term aberrant immune response to different triggers, or if the viral acute infection is just the first sign of a long-term airway morbidity related to host pre-existing susceptibility factors [2,3]. These perspectives are not mutually exclusive, being both relevant in recurrent wheeze development. Concerning susceptible host hypothesis, there is a growing interest in the study of how intrauterine environment, as well as perinatal and postnatal exposures, can influence respiratory and allergic diseases [4,5]. Longitudinal studies, focused on respiratory health prenatal and perinatal determinants, play a key role in the investigation of such cause-effect relationships. A recent birth cohort study demonstrated that early-life environmental exposures (such as maternal psychological state, tobacco smoke, allergens and microbes) show specific association with different childhood respiratory phenotypes, defined by wheezing and sensitization patterns [6].

Some studies investigated the role of single early life biomarkers as predictors of wheezing in children. Chawes et al. [7], for example, showed that exhaled nitric oxide in neonates was associated with early transient (but not persistent) wheezing.

Omic approaches may provide a different perspective on the investigation of the relationship between early life and the following respiratory health. In fact, being untargeted and not guided by any a-priori hypothesis about the underlying biological mechanisms, omic techniques may identify pattern of biomolecules associated with a condition of interest. In particular, metabolomics enables the study of the overall metabolic arrangement, a good indicator of organism’s phenotype, being metabolites the end-products of the system biology paradigm [8].

The most widely used spectroscopic techniques for metabolomics analysis are mass spectrometry (MS) (often combined with chromatographic separation) and Nuclear Magnetic Resonance (NMR) spectroscopy. Although MS system is less reproducible than NMR instrument, it allows a more sensitive detection of a major number of metabolites, useful for urine matrix that presents a wide range of compounds with a great variability in concentration [8,9].

Several biofluids have been analyzed through a metabolomic approach in studies investigating pediatric respiratory diseases [10]. The analysis of amniotic fluid samples enabled the discrimination of infants who are likely to present with wheezing in the first year of life [11]; the analysis of plasma samples [12] and exhaled breath condensate samples [13] enabled the discrimination of patients with severe asthma; the analysis of urine samples collected in children with recurrent wheezing enabled the discrimination of those with early-onset asthma [14]. Urine is a particularly suitable sample when working with young children, since it is easy to be collected non-invasively.

Aim of the present prospective study is to determine whether an untargeted metabolomic profiling of urine samples at birth may help in discovering a signature capable to identify children who will present with acute bronchiolitis (in the first year of life) and/or recurrent wheezing during the first 3 years of life.

## 2. Results

237 healthy newborns were enrolled at birth. 32 children were lost to follow-up, thus 205 subjects (132 males) were included in the analysis.

35 out of 205 infants (17%) had a pediatrician-based diagnosis of bronchiolitis (6 were hospitalized and 3 required oxygen supplementation), whereas 170 (83%) had no history of bronchiolitis in the first year of life. Among the 35 infants who had bronchiolitis, 11 had 3 or more episodes of wheezing during the 3-year follow-up. Within the group of 170 children that did not develop bronchiolitis, 41 had at least 1 subsequent episode of wheezing (13 had 3 or more episodes) and 129 had none. Then, bronchiolitis in the first year of life resulted to be a risk factor for wheezing (Fisher’s exact test *p* < 0.001 and power = 0.99). Respiratory Symptoms of the 205 children enrolled in the study are summarized in Table S1.

**Figure 1:**
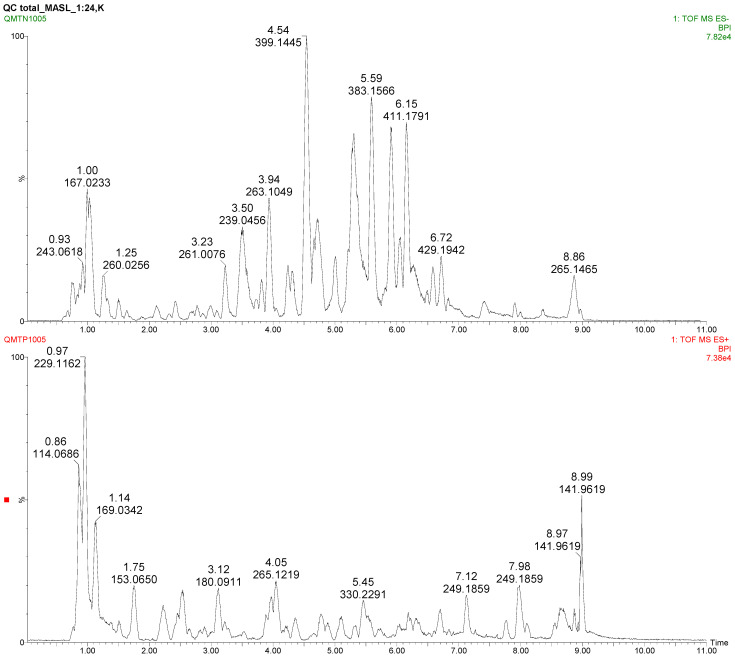
Chromatographic profiles (base peak chromatograms) of a Quality Control sample: negative ionization mode (**upper side**) and positive ionization mode (**lower side**).

With regard to metabolomics analysis, 131 metabolites were identified in the extracted data searching our in-house database based on standard compounds and MS/MS data. Identified metabolites belong to the families of steroids and steroid derivatives, alpha amino acids and derivatives, arginine and derivatives, beta amino acids and derivatives, bile acids, alcohols and derivatives, dicarboxylic acids and derivatives, indoles and derivatives, L-alpha-amino acids, nucleoside and nucleotide analogues, N-acyl-L-alpha-amino acids, organic acids, purines and purine derivatives, pyrimidine nucleosides and quinolines and derivatives (Table S2). We obtained a mass accuracy for the standard mix of less than 3 ppm for the 9 metabolites used, a change in retention times in a 0.20 min window and a coefficient of variation in ion intensity, both for the standards and for most of the identified metabolites in the QCs (85 metabolites), less than 20%, both in positive and in negative ionization mode. In Figure 1, the chromatographic profiles of a Quality Control sample acquired in negative and in positive ionization mode are shown.

After data pre-processing, the dataset obtained from the data acquired by positive ionization mode (POS dataset) showed 1196 Rt_mass variables and that arising from the data acquired by negative ionization mode (NEG dataset) included 1984 Rt_mass variables. The score scatter plots obtained considering the first two components of the PCA models of the two datasets are reported in Figure S1.

The PCA model of each subgroup of interest did not highlight the presence of outliers on the basis of the T2 (Hotelling T2 distance) and Q (distance to the model) test assuming α = 0.05.

### 2.1. Newborns with Bronchiolitis vs. Newborns without Bronchiolitis

**Figure 2:**
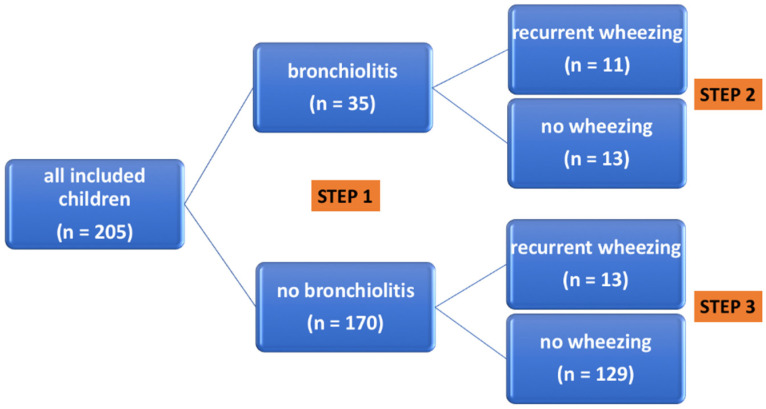
The three different steps in the analysis of our cohort of health neonates. Recurrent wheezing = at least 3 episodes of pediatrician-diagnosed wheezing.

As a first step of data analysis (Figure 2) the group of newborns that developed bronchiolitis was compared with the group without bronchiolitis.

The clinical variables of the mother-newborn pairs (metadata), investigated through univariate data and decision tree learning tools, demonstrated no relationships with the outcome bronchiolitis. CART showed the best performance in repeated cross-validation, but the obtained classification was not reliable (MCCcv = 0.19, *p* = 0.14). Among the risk factors (Table 1), only the male gender resulted significantly more frequent in children who developed bronchiolitis (Fisher’s exact test *p* = 0.012 and power = 0.78).

**Figure 3:**
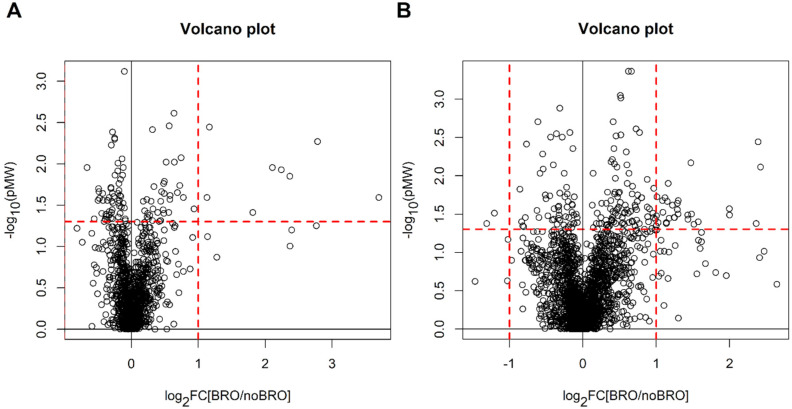
Newborns with bronchiolitis vs. newborns without bronchiolitis: Volcano plots obtained for the POS dataset (panel (**A**)) and for the NEG dataset (panel (**B**)); the dashed red lines indicate the limits of p Mann-Whitney (pMW) equal to 0.05 and |log_2_FC| = 1.

The metabolic profile of the collected urine samples was investigated by univariate and multivariate data analysis. Since the group of newborns without bronchiolitis was larger than that of newborns with bronchiolitis (170 vs. 35 subjects), we selected two subsets of 42 observations each that well-described the children without bronchiolitis. This selection was performed by Onion D-optimal design for the POS and the NEG datasets. The selected subsets were compared with the group of children with bronchiolitis. For the POS dataset, no differences have been observed by univariate analysis (q-values > 0.54 for all the features), and only weak classification models were obtained by multivariate analysis (MCCoob = 0.21 for PLS2C and MCCoob = 0.32 (*p* = 0.010) for RF). Similar results were obtained for the NEG dataset (q-values > 0.31 for all the features, MCCoob = 0.14 for PLS2C and MCCoob = 0.19 (*p* = 0.12) for RF). In Figure 3 are reported the Volcano plots obtained by univariate data analysis.

### 2.2. Children with Bronchiolitis with or without Recurrent Wheezing

As a second step of data analysis (Figure 2), within the groups of 35 children with bronchiolitis, we compared the group composed of children who presented at least 3 episodes of wheezing during follow-up (*n* = 11) (recurrent wheezers) and that of children who had none (*n* = 13).

The risk factor distribution was not different in these two subgroups (Table 1) and no reliable classification trees were generated on the basis of the collected metadata (both CART and conditional tree generated classifiers with MCCcv less than 0.10 and *p* > 0.30). At the level of single metadata, no differences have been observed.

Moreover, we found no reliable multivariate models capable of discriminating these two groups. For the POS dataset, the best PLS2C model showed MCCoob = 0.00 and the best RF MCC = 0.21 (*p* = 0.035) whereas, for the NEG dataset, the best PLS2C model showed MCCoob = 0.04 and the best RF MCCoob = 0.27 (*p* = 0.12).

**Figure 4:**
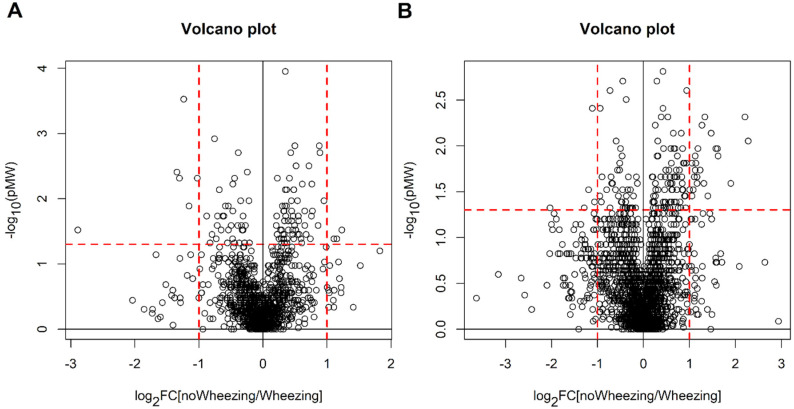
Children with bronchiolitis with or without recurrent wheezing: Volcano plots obtained for the POS dataset (panel (**A**)) and for the NEG dataset (panel (**B**)); the dashed red lines indicate the limits of p Mann-Whitney (pMW) equal to 0.05 and |log_2_FC| = 1.

Mann-Whitney test with false discovery rate did not discover relevant features (for the POS dataset, 2 variables showed q-value around 0.20 and the remaining features q-value greater than 0.30 whereas q-values resulted greater than 0.61 for all the features in the case of the NEG dataset). In Figure 4 are reported the Volcano plots obtained by univariate data analysis.

### 2.3. Children without Bronchiolitis with or without Recurrent Wheezing

In the third step of data analysis (Figure 2), the group of children that did not develop bronchiolitis was investigated. In this group of children (*n* = 170) the delivery mode resulted to be a risk factor for any wheezing: 36% of children born via caesarean delivery versus 18% of children born via vaginal delivery presented at least 1 episode of wheezing during the first 3 years of life (Fisher’s exact test *p* = 0.018 and power = 0.73).

Comparing children who had at least 3 episodes of wheezing (*n* = 13) with those who had none (*n* = 129), the risk factor distribution was not different in the two subgroups (Table 2) and the analysis of metadata using decision tree based techniques did not generate reliable models capable to predict recurrent wheezing. Specifically, both CART and conditional tree generated classifiers with MCCcv less than 0.05 (*p* > 0.20).

**Figure 5:**
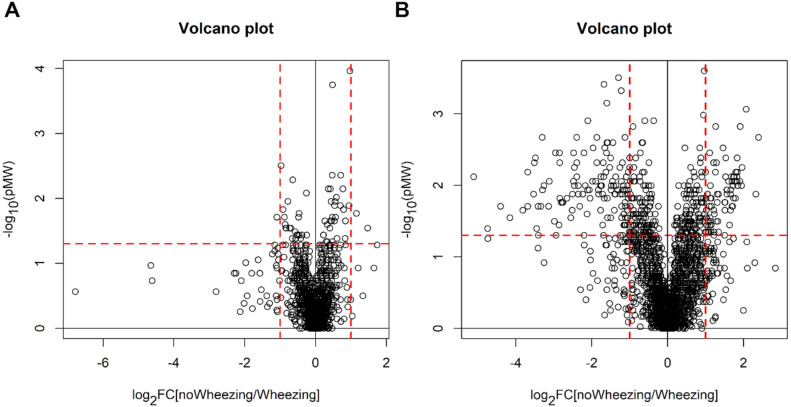
Children without bronchiolitis with or without recurrent wheezing: Volcano plots obtained for the POS dataset (panel (**A**)) and for the NEG dataset (panel (**B**)); the dashed red lines indicate the limits of p Mann-Whitney (pMW) equal to 0.05 and |log_2_FC| = 1.

Since the group of children without recurrent wheezing was larger than that of children with recurrent wheezing (129 vs. 13 subjects), a subset of 15 and a subset of 16 newborns without recurrent wheezing were selected by Onion D-optimal design considering the POS and the NEG dataset, respectively. The two subsets were compared with the group of children with wheezing. Interestingly, with the NEG dataset, both univariate and multivariate data analysis allowed us to discover a metabolic signature. Specifically, 201 variables showed q-values less than 0.10 and the best PLS2C model presented MCCoob = 0.46 and the best RF MCCoob = 0.48 (*p* = 0.019). The most interesting features were annotated finding that xanthine, uric acid, leucine, L-tyrosine, L-pyroglutamic acid and L-ornithine presented higher level in the group without wheezing than in the group with recurrent wheezing. In the case of the POS dataset, only weak classification models were obtained (MCCoob = 0.25 for PLS2C and MCCoob = 0.37 (*p* = 0.025)) and 2 variables showed q-values around 0.15 while the remaining features q-values greater than 0.80. In Figure 5 are reported the Volcano plots obtained by univariate data analysis.

## 3. Discussion

In this study, a population-based cohort study of term newborns, we found no metabolomic profiles at birth associated with the following occurrence of acute bronchiolitis and with the development of post-bronchiolitis recurrent wheezing.

On the other hand, we found an association between the urinary metabolomic profile at birth and the development, in the first 3 years of life, of recurrent wheezing not preceded by acute bronchiolitis.

### 3.1. Metabolomic Profile at Birth, Acute Bronchiolitis and Development of Post-Bronchiolitis Wheezing

We found no metabolomic profile at birth associated with the occurrence of acute bronchiolitis and with the development of post-bronchiolitis wheezing.

The association between acute viral bronchiolitis and wheezing is well known, and confirmed also by our data with a clear correlation between these two conditions. Nonetheless, it has long been debated whether acute bronchiolitis induces structural or immunological alterations which facilitate the recurrence of wheezing, or whether bronchiolitis and recurrent wheezing share common predisposing background. A recent metanalysis showed that, although a causal relationship between bronchiolitis and subsequent wheezing is plausible [15], such hypothesis could not be clearly confirmed [16].

In our study, the lack of an early metabolomic arrangement associated to the following occurrence of acute bronchiolitis was expected, while more interesting is the lack of a predisposing metabolomic background in both viral bronchiolitis and subsequent wheezing illness. This result stands against the existence at birth of an underlying biochemical-metabolic substrate predisposing to both acute bronchiolitis and post-bronchiolitis recurrent wheezing. On the other hand, we cannot exclude that bronchiolitis and the following recurrent wheezing share a susceptibility related to genetic background [16] or to a pre-existing reduced lung function [17,18]. Even though, a recent longitudinal study (in 1143 infants) showed that lung function impairments after bronchiolitis were independent of baseline lung function, suggesting that the risk of recurrent wheezing does not reflect underlying lung structural or functional deficiencies [19].

To the best of our knowledge, this is the first study investigating the possible role of a metabolomic approach applied at birth (before symptom onset) in the prediction of post-bronchiolitis wheezing. Other studies explored metabolomic changes induced by acute bronchiolitis and potentially related to the subsequent wheezing illness [20,21]. One of our previous studies, applying the same approach used in the present one, described the specific metabolomic profile in infants with acute viral bronchiolitis who later develop recurrent wheezing [20]. Altogether, our studies suggest that metabolomic analysis is unable to identify at birth a profile predictive of post-bronchiolitis wheezing, but it can capture the early metabolic disruption caused by bronchiolitis and associated with the following wheezing illness.

### 3.2. Metabolomic Profile at Birth and Development of Recurrent Wheezing Not Preceded by Bronchiolitis

In order to remove the possible influence of bronchiolitis on recurrent wheezing pathogenesis, we excluded children who had acute bronchiolitis. Among children with no history of bronchiolitis, we compared those with and without recurrent wheezing. Interestingly, this comparison enabled the discrimination at birth of the neonates who will develop recurrent wheezing from those who will not.

In particular, children who do not develop recurrent wheezing in the first 3 years of life are characterized by increased urinary levels at birth of a number of metabolites with a chemical structure ascribable to xanthine, uric acid and some amino acids or their derivatives (leucine, L-tyrosine, L-ornithine and L-pyroglutamic acid). At the moment, we cannot suggest a pathophysiological connection between these metabolites and the evaluated clinical outcome, as well as we cannot establish whether the increased level of these metabolites is protective or, on the contrary, whether their reduction represents a risk factor for recurrent wheezing. For example, reduced levels of a tyrosine metabolite have been previously reported in infants who later develop asthma [22]. Regardless the specific pathogenetic role of the identified metabolites, in our opinion, it is definitely interesting the finding of a metabolic arrangement, already present at birth, associated with the development of recurrent wheezing not triggered by bronchiolitis.

In keeping with our data, increased levels of exhaled nitric oxide, a marker of eosinophilic airway inflammation, were reported in high risk neonates (born to mothers with asthma) who develop transient (but not persistent) wheezing [7], suggesting a possible role for early inflammation in wheezing illness pathogenesis. Moreover, a metabolomic fingerprint associated with the subsequent development of asthma was described in urine samples collected at one month of age in children born to mothers with asthma [23].

### 3.3. Limits and Strengths of the Study

Our study has some limitations. From a clinical standpoint, a first limit is the lack of data on the etiological agents of acute bronchiolitis. Nonetheless, we considered bronchiolitis diagnosed by a pediatrician, accordingly to a strict clinical definition.

A second limitation is that only one chromatographic method was applied, limiting the description of the whole urine metabolome. We cannot exclude that the use of other separation methods could discover other differences in the investigated samples.

Our study also has some significant strengths. First, we recruited and closely followed a well-characterized cohort of newborn, with a relatively low number of subjects lost at follow-up. Second, we applied a reference UPLC-MS based analytical method for metabolomic analysis and a well-established multivariate statistical approach, which in previous studies already enabled a clear discrimination between groups under investigation [14,20].

## 4. Materials and Methods

### 4.1. Study Population and Study Design

This was a prospective longitudinal study, conducted in a cohort of healthy newborns born at term (37–42 Gestational Weeks, birth weight >2500 g) at the Department of Women’s and Children’s Health of Padova, between February and November 2013. Exclusion criteria were: distress at birth, early-onset sepsis, need for any medical therapy, any metabolic disorder (e.g., hypoglycemia), any major congenital organ abnormality.

Relevant clinical variables of the mother-newborn pairs were registered (metadata). For the mother: ethnicity, pregnancy related conditions, gestational weight gain, vaginal swab test results, smoking during pregnancy; for the newborn: gender, gestational week at birth, birth weight, Apgar score, mode of delivery.

After birth (mean age 2 days (SD 0.9)) a urine sample was collected in sterile polypropylene containers previously washed with methanol, and 1 aliquot of 3 mL volume stored at −80 °C until metabolomic analysis.

Children were followed for 3 years, being evaluated at 6, 12, 24 and 36 months. At each time point, history was taken using a locally-developed questionnaire, focused on respiratory and allergic symptoms.

At the end of the first year of life children were divided in two groups: those who had developed an acute episode of bronchiolitis and those who had not. Acute bronchiolitis was defined as the first episode of lower respiratory tract infection with signs of respiratory distress and fine crackles all over the chest, preceded by a coryzal illness, as reported by a pediatrician [24].

At the end of the third year of life children were classified as recurrent wheezers if they reported at least 3 episodes of pediatrician-diagnosed wheezing.

The study was approved by the ethical review boards of our Hospital (protocol number 2860P, approved on 13 May 2013) and all children’s parents gave their written informed consent.

### 4.2. Metabolomic Analysis

#### 4.2.1. Chemicals and Reagents

For the UPLC-MS analysis, high-purity MS-grade solvents were used. Formic acid (FA), methanol, and acetonitrile were obtained from Sigma-Aldrich (2021 Merck KGaA, Darmstadt, Germany) and used without further purification. Water was purified with a Milli-Q Elix purification system (Millipore, Bedford, MA, USA).

#### 4.2.2. Urine Analysis

The analysis was performed at the Laboratory of Mass Spectrometry and Metabolomics of the Department of Women’s and Children’s Health, University of Padua’s.

Urine samples were slowly thawed overnight at +4 °C and then transferred to ambient temperature for the preparation. Each sample was stirred and centrifuged at 3600× *g* for 10 min at 10 °C, then 50 µL of the supernatant from each sample were pipetted in a total recovery glass vial, adding 100 µL of 0.1% FA solution in water (final dilution 1:3).

Untargeted metabolic profiling of urine samples was performed in positive and negative electrospray ionization (ESI) mode on an Acquity Ultra Performance Liquid Chromatography (UPLC) system (Waters, UK) coupled to a Quadrupole Time-of-Flight (QToF) Synapt G2 HDMS mass spectrometer (Waters MS Technologies, Ltd., Manchester, UK). Chromatography was performed using an Acquity HSS T3 (1.7 μm, 2.1 × 100 mm) column (Waters Corporation, Milford, CT, USA) kept at 50 °C. The flow rate of the mobile phase was set at 0.5 mL/min, and each sample run lasted 11 min, with 5 µL of the sample injected for each run. For mass accuracy, a LockSpray interface was used with a 20 μg/L leucine enkephalin. Data were collected in continuum (profile) mode, in a scanning range of 20–1200 *m*/*z*, with acquisition rate of 0.3 s, and with lock mass scans collected every 10 s and averaged over 3 scans for mass correction.

The gradient mobile phase consisted of water with 0.1% FA (A) and methanol with acetonitrile in a 90:10 ratio with 0.1% FA (B). Each sample run lasted 11 min and of an isocratic phase of 5% B for 1 min, a linear increase to 30% B in 2.5 min, a linear increase to 95% B in 3 min, an isocratic phase of 95% B for 1.5 min, a washout phase of 5% B for 3 min.

Quality Control samples (QC), blank samples (Blank) and Standards Solution Samples (Mix) were used to monitor the performance during the analytical session. The QCs were prepared from an aliquot (25 µL) of each sample, pooled together and diluted 1:3 with 0.1% formic acid solution in water, treated as the samples. The Mix consisted of nine compounds of known exact mass and retention time. The name, the retention time, the neutral mass and the concentration of the standards used were reported in Table S3 of the Supplementary Materials.

The QCs, Blanks and Mixes were injected at regular intervals of 12 samples during the sequence, to identify specific ions from the mobile phase, and any contaminants. The sequence for the analysis were prebuilt in Excel to randomize the samples injections and prevent any spurious classification deriving from the position of the sample in the sequence. Data were pre-processed using Progenesis QI software (Waters Corporation, Milford, CT, USA). The parameters used for data extraction were optimized through the preliminary processing of the QCs. We set a filter of 0.5 and 0.25 to import the raw data, respectively for positive and negative ionization mode, and we used a sensitivity of 3 for the automatic peak picking, in a chromatographic range from 0.4 to 8.0 min. Features with at least a missing data in the QCs were excluded. The resulting set of features was filtered excluding all the features with a ratio between 95th percentile in the Blanks and 5th percentile in the QCs greater than 0.20 and a coefficient of variation greater than 20% for the QCs. Missing data were imputed generating a random number between zero and the minimum measured value of the feature. Probabilistic quotient normalization (PQN) was used to remove effects of dilution on sample concentration (18) and to reduce the loss of signal intensity due to intra-batch effect. After data normalization, features showing a CV greater than 20% in the QCs were excluded. The number of features after each step of data pre-processing are reported in Table S4. Data were log-transformed and mean-centered prior to performing multivariate data analysis.

#### 4.2.3. Statistical Data Analysis

Metadata were investigated by decision tree learning tools (19). Conditional tree and Classification and Regression Tree (CART) have been applied to discover schemes of decision rules capable to distinguish the groups of interest. Model parameters have been optimized by 50-repeated 5-fold cross-validation. To investigate the relationships between metadata and groups of interest at the level of single metadata, Fisher’s exact test was applied in the case of categorical variables whereas *t*-test and Mann-Whitney test have been applied in the case of normally and non-normally distributed continuous data, respectively. Normality was assessed using the Shapiro-Wilk test assuming normally distributed data for *p* > 0.10. A significant level α = 0.05 has been assumed. Performance in classification was measured calculating the Matthew correlation coefficient (MCC) by 50-repeated 5-fold cross-validation (MCCcv) for conditional tree and CART. Permutation test on the class response (1000 random permutations) was applied to estimate p of the parameters used to measure the performance of the classifiers.

Metabolomic data were investigated both by univariate data analysis and by multivariate techniques. Specifically, fold change (FC) and Mann-Whitney test controlling the false discovery rate by Storey’s method were applied to discover perturbation at the level of single feature whereas Principal Component Analysis (PCA), Partial Least Squares for classification (PLS2C) (20) and Random Forest (RF) were used to evaluate the cooperative effects of the measured features. Stability selection was applied to PLS2C (200 sub-models were generated performing variable selection based on VIP and the best MCCcv) to select the most relevant features and to estimate the performance in prediction of the model through the calculation of MCC using the out-of-bag prediction (MCCoob). The number of score components was assessed on the basis of the first maximum of MCCcv. The performance in prediction of RF was estimated by MCCoob. Permutation test on the class response (1000 random permutations) was applied to estimate p of the parameters used to measure the performance of the classifiers. The parameters of the best PLS2C models and RFs are reported in Tables S5 and S6 of Supplementary Materials, respectively. Since unbalanced classes may introduce bias in the classifier, we selected a subset of the largest class applying Onion D-optimal design (21) to the candidate set described using the PCA-scores and Q calculated using the metabolomics data in order to have balanced classes. Onion D-optimal design assures that the selected samples are representative of the whole candidate set, without loss of the peculiarities of the set. The number of principal component to use was determined as the minimum number to obtain a PCA model with explained variance at least of 60% of the total variance.

Data analysis has been performed by in house R-functions implemented using the R 4.0.4 platform (R Foundation for Statistical Computing, Vienna, Austria). Packages “rpart” and “party” have been used to generate CART and conditional tree, respectively, while RF has been built using the “randomForest” package.

## 5. Conclusions

In conclusion, through a well-established untargeted metabolomic approach, we found no biochemical-metabolic dysregulation at birth associated with the following occurrence of acute bronchiolitis. Moreover, we found no metabolomic profile at birth associated with the development of post-bronchiolitis recurrent wheezing in the first 3 years of life. These findings do not support the existence of an underlying susceptibility background for bronchiolitis and post-bronchiolitis recurrent wheezing.

On the other hand, a metabolomic urinary signature was described at birth associated with the development of recurrent wheezing not preceded by acute bronchiolitis, suggesting that the early biochemical-metabolic arrangement could put some children at risk of developing this condition. Further studies are needed to better investigate the pathophysiological role of the identified metabolites and to understand how such metabolic background interacts with other known risk factors in the pathogenesis of recurrent wheezing.

## Figures and Tables

**Table 1:** Risk factors distribution in children according to the development of bronchiolitis and post bronchiolitis recurrent wheezing.

	Bronchiolitis (*n* = 35)	No Bronchiolitis (*n* = 170)	*p*	Post-Bronchiolitis Recurrent Wheezing (*n* = 11)	No Post-Bronchiolitis Wheezing (*n* = 13)	*p*
Males	29 (83%)	103 (61%)	0.012	10 (91%)	9 (69%)	0.327
Atopic parents	17 (49%)	81 (48%)	1.000	5 (45%)	6 (46%)	1.000
Smoke exposure	9 (26%)	70 (41%)	0.126	3 (27%)	3 (23%)	1.000
Vitamin D supplementation	27 (77%)	135 (79%)	0.470	6 (55%)	6 (46%)	1.000
Exclusive breast feeding for 6 months	12 (34%)	51 (30%)	0.454	4 (36%)	4 (31%)	1.000
Siblings	26 (74%)	108 (64%)	0.248	10 (91%)	11 (85%)	1.000
Pets in the house	14 (40%)	70 (41%)	0.849	3 (27%)	8 (62%)	0.123
Mold in the house	20 (57%)	71 (42%)	0.135	8 (73%)	5 (38%)	0.123
Dust mite exposure	30 (86%)	152 (89%)	0.557	10 (91%)	11 (85%)	1.000
Hospitalization for bronchiolitis	6 (17%)	--	--	3 (27%)	3 (23%)	1.000

**Table 2:** Risk factors distribution in children according to the development of recurrent wheezing in the no-bronchiolitis group.

	Recurrent Wheezing (*n* = 13)	No Wheezing (*n* = 129)	*p*
Males	5 (38%)	80 (62%)	0.138
Atopic parents	8 (62%)	63 (49%)	0.562
Smoke exposure	8 (62%)	50 (39%)	0.142
Vitamin D supplementation	11 (85%)	111 (86%)	1.000
Exclusive breast feeding for 6 months	3 (23%)	47 (36%)	0.543
Siblings	7 (54%)	83 (64%)	0.549
Pets in the house	8 (62%)	53 (41%)	0.239
Mold in the house	4 (31%)	57 (44%)	0.396
Dust mite exposure	10 (77%)	117 (91%)	0.142

## Data Availability

The data presented in this study are available (anonymized) on request from the corresponding author and online.

## Supplementary Materials

The following are available online at https://www.mdpi.com/article/10.3390/metabo11120825/s1, Figure S1. Score scatter plots of the PCA models obtained considering the POS dataset (panel A) and the NEG dataset (panel B). Table S1. Respiratory Symptoms of the 205 children enrolled in the study. Table S2. 156 Variables identified in ESI+ and ESI− searching our in-house database, corresponding to 131 metabolites. Table S3. Compounds of the Standard Mix. Table S4. Number of features after each step of data pre-processing. Table S5. Parameters of the best PLS for classification model obtained for untargeted metabolomics data. Table S6. Parameters of the best RF obtained for untargeted metabolomics data.
