# Supplementary material for: Metabolomic Profile at Birth, Bronchiolitis and Recurrent Wheezing: A 3-Year Prospective Study
Source: Metabolites. 2021 Nov 30;11(12):825. doi: 10.3390/metabo11120825 (PMC8706329; doi:10.3390/metabo11120825)
Supplement: Supplementary file 1 [file metabolites-11-00825-s001.zip › Supplementary_Materials_MS.pdf]

SUPPLEMENTARY MATERIALS

Figure S1. Score scatter plots of the PCA models obtained considering the POS dataset (panel A) and the NEG dataset (panel B). The source of data variation associated to the analytical session (represented by the QCs reported with red circles) is negligible when compared to the biological variability of the urine samples. Samples are represented using different symbols and colors according to their group (see legend).

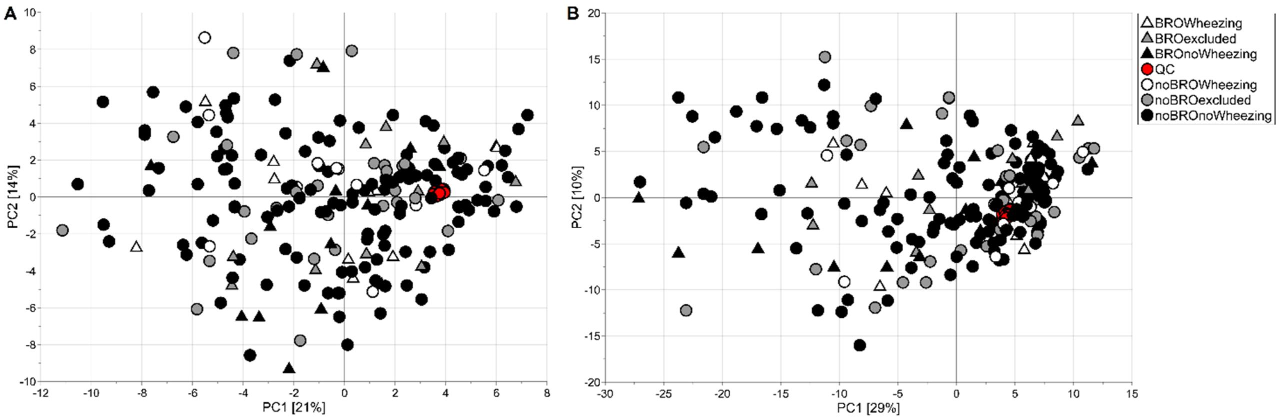

Table S1. Respiratory Symptoms of the 205 children enrolled in the study.

	**Number of children (%)**
Bronchiolitis in the first year of life	35 (17%)
No episodes of wheezing	142 (69,2%)
Wheezing (1 or 2 episodes) in the first 3 years of life	39 (19%)
Wheezing (3 or more episodes) in the first 3 years of life	24 (11,7%)

Table S3. Compounds of the Standard Mix.

**description**	**Retention time [min]**	**Neutral mass**	**concentration [µM]**
Sulfaguanidine	0.98	214.05252	37
Acetaminophen	2.62	151.06342	33
Hippuric acid	3.61	179.05882	120
Caffeine	3.92	194.08042	25
Leucine Enkephalin	4.95	555.26932	0.90
Sulfadimethoxine	5.00	310.07362	4.0
Verapamil	5.41	454.28322	0.40
Terfenadine	6.00	471.31382	2.0
Cholic Acid	6.99	408.286976	0.60

Table S4. Number of features after each step of data pre-processing.

	**positive ionization mode**	**negative ionization mode**
extracted features	6849	5709
**filter**		
missing values in QCs	6702	5491
95° BLK/5° QC>0.20	4907	4473
CV QC>20%	1204	2047
CV QC after PQN<20%	1196	1984

Table S5. Parameters of the best PLS for classification model obtained for untargeted metabolomics data; A is the number of latent variables, N features is the number of relevant features, MCCcv is the Matthew correlation coefficient calculated by 50-repeated 5-fold cross-validation and MCCoob is the median MCC calculated considering the out-of-bag observations during stability selection.

**model**	**dataset**	**A**	**N features**	**MCC**	**MCCcv**	**MCCoob**
bronchiolitis (N=35) vs no bronchiolitis (N=42)	POS	1	98	0.28 (0.42)	0.25 (0.010)	0.21
bronchiolitis (N=35) vs no bronchiolitis (N=42)	NEG	1	176	0.27 (0.36)	0.22 (0.040)	0.14
bronchiolitis: recurrent wheezing (N=11) vs no wheezing (N=13)	POS	1	78	0.77 (0.010)	0.44 (0.035)	0.00
bronchiolitis: recurrent wheezing (N=11) vs no wheezing (N=13)	NEG	1	139	0.17 (0.96)	0.09 (0.38)	0.04
no bronchiolitis: recurrent wheezing (N=13) vs no wheezing (N=15)	POS	1	84	0.57 (0.082)	0.36 (0.060)	0.25
no bronchiolitis: recurrent wheezing (N=13) vs no wheezing (N=16)	NEG	1	185	0.53 (0.066)	0.48 (0.022)	0.46

Table S6. Parameters of the best RF obtained for untargeted metabolomics data; ntree is the number of trees of the forest, mtry is the number of variables randomly sampled as candidates at each split, node size is minimum size of terminal nodes, MCCoob is the Matthew correlation coefficient calculated by out-of-bag prediction.

**model**	**dataset**	**mtry**	**ntree**	**node size**	**MCC**	**MCCoob**
bronchiolitis (N=35) vs no bronchiolitis (N=42)	POS	69	400	5	1.00 (0.99)	0.32 (0.010)
bronchiolitis (N=35) vs no bronchiolitis (N=42)	NEG	72	1000	18	0.90 (0.98)	0.19 (0.12)
bronchiolitis: recurrent wheezing (N=11) vs no wheezing (N=13)	POS	56	200	4	1.00 (0.99)	0.21 (0.035)
bronchiolitis: recurrent wheezing (N=11) vs no wheezing (N=13)	NEG	89	400	6	1.00 (0.99)	0.27 (0.12)
no bronchiolitis: recurrent wheezing (N=13) vs no wheezing (N=15)	POS	30	400	2	1.00 (0.99)	0.37 (0.025)
no bronchiolitis: recurrent wheezing (N=13) vs no wheezing (N=16)	NEG	45	1000	4	1.00 (0.99)	0.48 (0.019)
